# *Lenomyrmex hoelldobleri*: a new ant species discovered in the stomach of the dendrobatid poison frog, *Oophaga sylvatica* (Funkhouser)

**DOI:** 10.3897/zookeys.618.9692

**Published:** 2016-09-19

**Authors:** Christian Rabeling, Jeffrey Sosa-Calvo, Lauren A. O'Connell, Luis A. Coloma, Fernando Fernández

**Affiliations:** 1 Department of Biology, University of Rochester, Rochester, NY 14627, USA; 2 FAS Center for Systems Biology, Harvard University, Cambridge, MA 02138, USA; 3 Centro Jambatu de Investigación y Conservación de Anfibios, Fundación Otonga, San Rafael, Quito, Ecuador; 4 Universidad Regional Amazónica Ikiam, Muyuna, Tena, Ecuador; 5 Instituto de Ciencias Naturales, Universidad Nacional de Colombia, Apartado 7495, Bogotá D.C., Colombia

**Keywords:** Formicidae, Dendrobatidae, feeding ecology, myrmecophagy, cryptic species

## Abstract

The ant genus *Lenomyrmex* was recently discovered and described from mid to high elevation rainforests in southern Central and northwestern South America. *Lenomyrmex* currently consists of six described species, which are only rarely collected. Here, we add a new species, *Lenomyrmex hoelldobleri* **sp. n.**, which was discovered in a stomach content sample of the dendrobatid frog, *Oophaga sylvatica*, from northwestern Ecuador. *Lenomyrmex hoelldobleri* can be distinguished from other species in the genus by the presence of a well-developed petiolar node, whereas in all other species the node of the petiole is ill-defined. In addition to the shape of the petiolar node, *Lenomyrmex hoelldobleri* can be distinguished from the morphologically similar *Lenomyrmex costatus* by (i) the presence of the metanotal suture, (ii) the direction of the striae on dorsum of propodeum (concentrically transverse in *Lenomyrmex hoelldobleri*, longitudinal in *Lenomyrmex costatus*), (iii) the finely striate dorsum of postpetiole, (iv) its larger size, and (v) distinctly darker coloration. We also describe the gyne of *Lenomyrmex foveolatus*. This collection record from northwestern Ecuador extends the geographic distribution of *Lenomyrmex foveolatus* 400 km south from its previous record in Colombia. A revised taxonomic key to the workers and gynes of all described *Lenomyrmex* species is provided. We discuss the taxonomic relationship of *Lenomyrmex hoelldobleri* to other species in the genus and its biology based on the limited information that is currently available. Finally, we briefly discuss the feeding ecology of dendrobatid poison frogs in the context of providing a valuable source of rarely collected and cryptic new ant species.

## Abstract (es)

El género de hormigas *Lenomyrmex* fue recientemente descubierto y descrito de bosques lluviosos tropicales de mediana a gran altitud en el sur de Centro América y del noroeste de Sur América. El género *Lenomyrmex* está actualmente compuesto de seis especies, las cuales son raramente colectadas. En este artículo, agregamos una especie nueva, *Lenomyrmex hoelldobleri* **sp. n**., que fue descubierta en una muestra de contenido estomacal de la rana dendrobátida, *Oophaga sylvatica*, colectada en el noroeste de Ecuador. *Lenomyrmex hoelldobleri* se puede distinguir de las otras especies del género por la presencia del nodo del pecíolo bien desarrollado, mientras que en todas las demás especies del género el nodo del pecíolo está mal definido o ausente. Además de la forma del nodo peciolar, *Lenomyrmex hoelldobleri* se puede distinguir de *Lenomyrmex costatus* por (i) la presencia de la sutura metanotal, (ii) la dirección de las estrías en el dorso del propodeo (concéntricamente transversal, en *Lenomyrmex hoelldobleri*, longitudinal en *Lenomyrmex costatus*), (iii) el dorso del postpecíolo finamente estriado, (iv) su mayor tamaño, y (v) la coloración más oscura. También se describe la reina de la especie *Lenomyrmex foveolatus*. Esta colección del noroeste de Ecuador amplía la distribución geográfica de *Lenomyrmex foveolatus* 400 kilómetros al sur de su registro previo en Colombia. Se presenta una clave taxonómica revisada para las obreras y reinas de todas las especies descritas de *Lenomyrmex*. Se discute la relación taxonómica de *Lenomyrmex hoelldobleri* con otras especies del género y su biología con base a la información limitada que está disponible actualmente. Finalmente, discutimos brevemente la ecología de la alimentación de las ranas venenosas dendrobátidas en el contexto de ser una valiosa fuente de especies de hormigas crípticas, nuevas y raramente recolectadas.

## Introduction

The subfamily Myrmicinae is the most diverse clade of ants with currently more than 6,600 species, which is roughly equivalent to half the number of all described ant species (Bolton 2016). Within the past two decades ten new myrmicine genera and many more species have been discovered and described from the New World, including the extant genera *Cryptomyrmex*, *Cyatta*, *Diaphoromyrma*, *Dolopomyrmex*, *Kalathomyrmex*, *Kempfidris*, *Lenomyrmex*, *Mycetagroicus*, *Patagonomyrmex*, and *Tropidomyrmex*, testifying to the enormous diversity of this ant subfamily (Sosa-Calvo et al. 2013, and references therein; Fernández et al. 2014, Johnson and Moreau 2016). The myrmicine ants likely originated some 100 Million years ago during the late Cretaceous and the species in this group dispersed to all major ecosystems around the world (Ward et al. 2015). In addition to their hyperdiversity, vast geographic distribution, and old age of the clade, myrmicine ants also occupy diverse ecological niches (Hölldobler and Wilson 1990). Generalist predators and scavengers are common in speciose genera, such as *Crematogaster*, *Monomorium*, *Myrmica*, *Pheidole*, *Solenopsis*, and *Tetramorium*. In addition, highly specialized feeding habits originated in multiple myrmicine clades during the Paleocene and potentially contributed to the species richness and ecological success of these lineages. Especially noteworthy are the intricate behaviors of the seed harvesting ants, the fungus-growing ants, and the highly predaceous dacetine ants, which were expertly reviewed in Hölldobler and Wilson's (1990) landmark monograph “The Ants”.

Just prior to the turn of the millennium, Fernández and Palacio (1999) described the myrmicine genus *Lenomyrmex* from the Neotropical region. *Lenomyrmex* ants are rarely collected and seven species are currently known from this genus, including *Lenomyrmex hoelldobleri* sp. n., the species described here. The geographic distribution of *Lenomyrmex* extends from Costa Rica in the North to southwestern Ecuador in the South, and only *Lenomyrmex inusitatus* is found on the eastern slope of the Andes (Fernández and Palacio 1999, Fernández 2001, Fernández and Sendoya 2004, Longino 2006, Delsinne and Fernández 2012). So far, all *Lenomyrmex* species were found in moist tropical rainforests, associated with medium and high elevation between 500 and 1800 meters above sea level (Longino 2006, Delsinne and Fernández 2012). The slender, elongate, and highly conspicuous mandibles with minute peg-like denticles are a synapomorphy of all *Lenomyrmex* species, suggesting specialized predatory habits (Fernández and Palacio 1999). Unfortunately, the feeding behavior of these rather cryptic ants was never observed and the prey organisms *Lenomyrmex* feeds on are unknown. *Lenomyrmex* appears to be a close relative of *Daceton* trap-jaw ants, which are both part of a monophyletic group of specialized predators (Ward et al. 2015).

**Figure 1.**
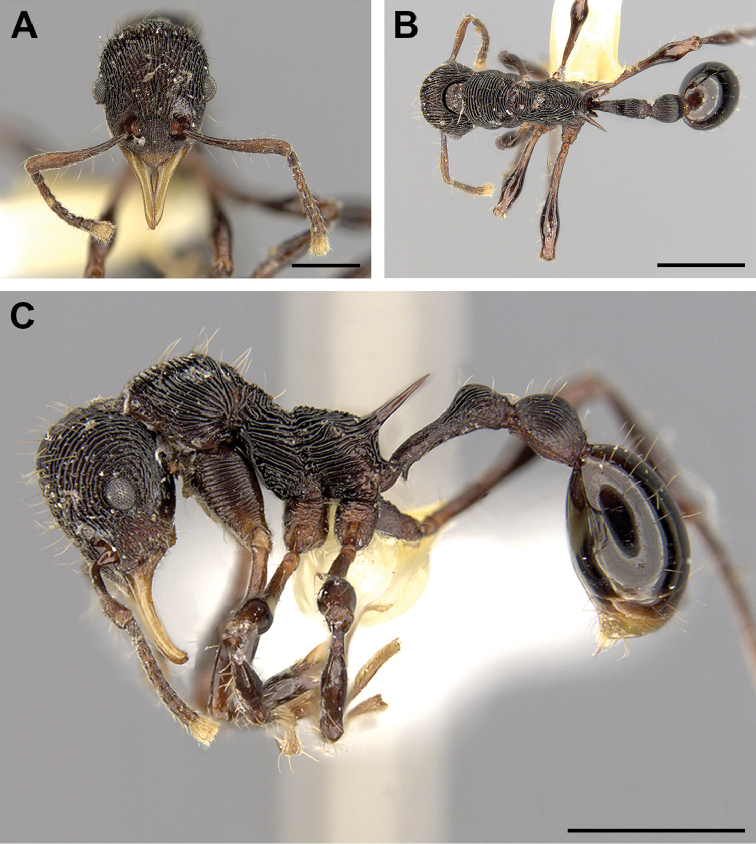
Worker of *Lenomyrmex hoelldobleri* in full-face (**A**), dorsal (**B**), and lateral (**C**) views. The depicted worker is the holotype with the unique specimen identifier USNMENT01124322. Scale bars: 0.5 mm (**A**), 1 mm (**B, C**).

Here, we describe the new species *Lenomyrmex hoelldobleri* sp. n. from northwest Ecuador (Fig. 1), which was discovered in stomach content samples of the dendrobatid poison frog, *Oophaga sylvatica*. We also diagnose the gyne of *Lenomyrmex foveolatus* (Fig. 3), which also fell prey to *Oophaga sylvatica*. This new record of *Lenomyrmex foveolatus* from northwest Ecuador expands the known geographic distribution range of this species from Colombia to Ecuador (Fig. 4). Many amphibians, including species of the aposematic poison frogs in the family Dendrobatidae, and non-avian reptiles are known to be specialized predators of ants (Weber 1938, Darst et al. 2005, Esteves et al. 2008, Sosa-Calvo 2015), and therefore they provide interesting sources of rarely collected and new arthropod species. Dendrobatid poison frogs sequester alkaloids that are found in their skin toxins from their diet (Daly et al. 2000, Saporito et al. 2004, 2007, Darst et al. 2005, McGugan et al. 2016), and therefore we briefly discuss the ecology of the specialized ant feeding behavior, or myrmecophagy, of dendrobatid frogs.

**Figure 2.**
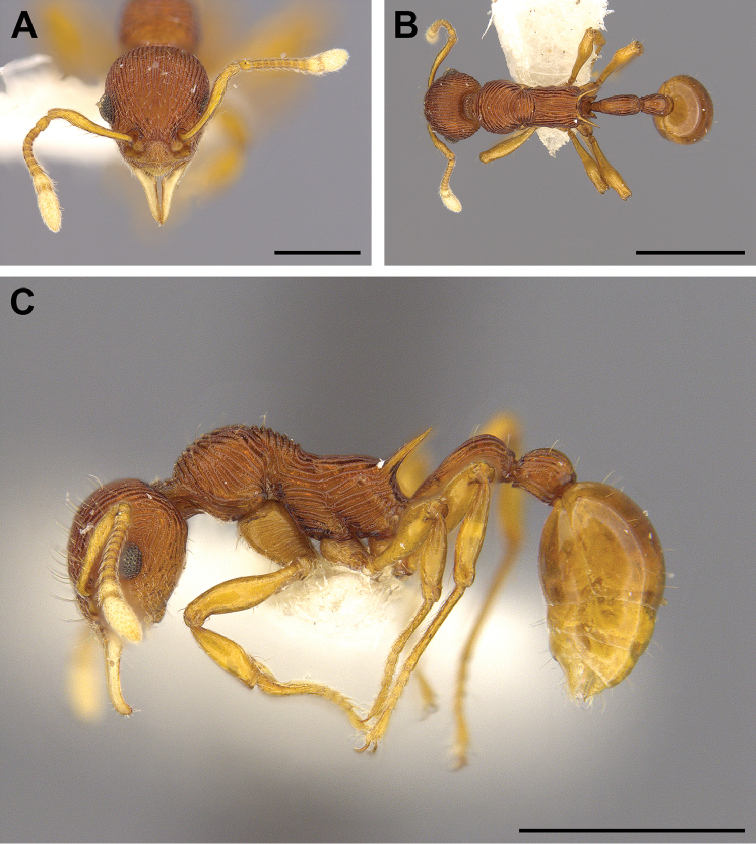
Worker of *Lenomyrmex costatus* in full-face (**A**), dorsal (**B**), and lateral (**C**) views. The depicted worker is the holotype with the unique specimen identifier MCZ-ENT00036069. Scale bars: 0.5 mm (**A**), 1 mm (**B, C**).

**Figure 3.**
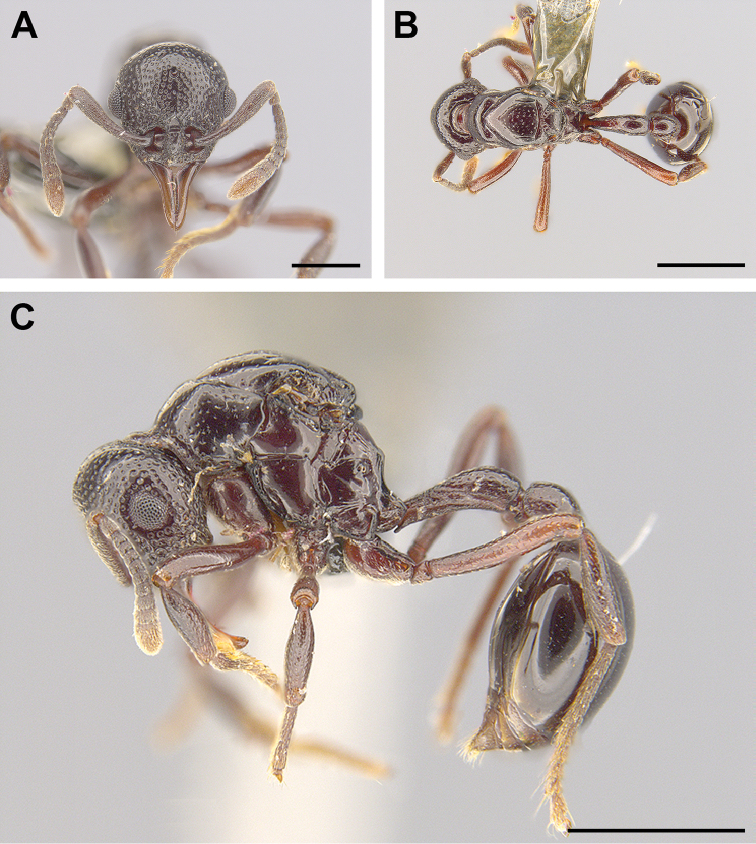
Dealate gyne of *Lenomyrmex foveolatus* in full-face (**A**), dorsal (**B**), and lateral (**C**) views. The depicted gyne has the unique specimen identifier USNMENT01127956. Scale bars: 0.5 mm (**A**), 1 mm (**B, C**).

**Figure 4.**
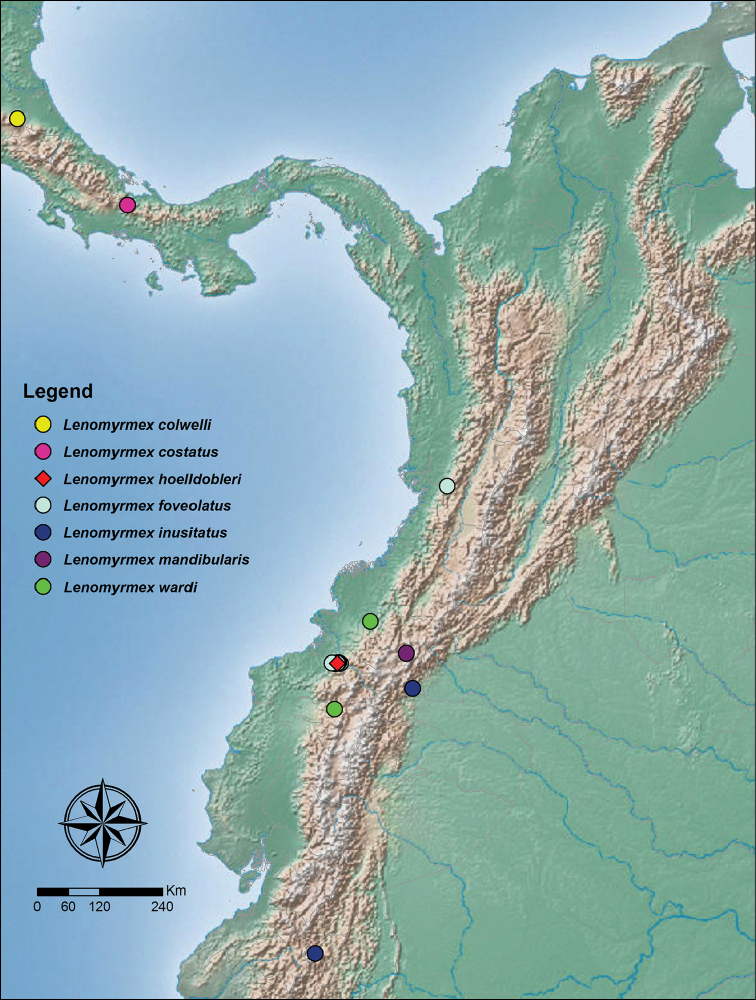
Geographic distribution of the genus *Lenomyrmex* in Central and South America.

## Materials and methods

**Material examined.** The examined ant and frog specimens have been deposited at the following institutions.

CJ
Centro Jambatu de Investigación y Conservación de Anfibios, Fundación Otonga, Quito, Ecuador 


CRC
 Christian Rabeling Collection, University of Rochester, Rochester, NY, U.S.A. 


DZUP
 Coleção Entomológica Pe. Jesus Santiago Moure, Universidade Federal do Paraná, Curitiba, PR, Brazil 


ICN
 Instituto de Ciencias Naturales, Universidad Nacional de Colombia, Bogotá D.C., Colombia 


MCZC
Museum of Comparative Zoology, Harvard University, Cambridge, MA, U.S.A. 


QCAZ
 Museo de Zoología, Pontificia Universidad Católica del Ecuador, Quito, Ecuador 


USNM
 United States National Museum of Natural History, Washington, DC, U.S.A. 


**Morphological analysis.** Specimens were examined and measured using a Leica M165 C stereomicroscope fitted with a stage micrometer. Measurements were recorded to the nearest 0.01 mm at 40x magnification. To generate composite images of the specimens we utilized a Leica DFC450 digital camera mounted on a Leica M205 C stereomicroscope. Composite images were assembled using the Leica Application Suite (Version 4.5) and the Helicon Focus (Version 6.2.2) software packages. Conventions for morphological terminology, measurements, and indices follow those utilized in recent taxonomic studies of Neotropical ants and frogs (Fernández and Palacio 1999, Brown et al. 2011, Delsinne and Fernández 2012, Rabeling et al. 2015). Measurements are given in millimeters. Measurements and indices are defined as follows:

EL
 Eye length, in lateral view, the maximum diameter of the eye 


GL
 Gaster length, in lateral view, from the anterior edge of the first tergum to the posterior edge of the last visible tergum 


HL
 Head length, in full-face view, the maximum distance from the anterior margin of the clypeus to the posterior margin of the head, excluding the mandibles 


HW
 Head width, in full-face view, the maximum width of the head excluding the compound eyes 


ML
 Mandible length, in full-face view, the maximum distance from the anterior margin of clypeus to the distalmost margin of the mandibles 


PL
 Petiole length, in lateral view, the axial distance from anteriormost margin of the ventral process to the posteriormost margin of petiole 


PPL
 Postpetiole length, in lateral view, the maximum axial distance from the anteriormost to the posteriormost margin of the postpetiole 


PPW
 Postpetiole width, in dorsal view, the maximum transverse distance across the disc of the postpetiole 


PW
 Petiole width, in dorsal view, the maximum transverse distance across the node 


SL
 Scape length, maximum length excluding the basal condyle 


SVL
 Snout to vent length, in ventral view, from the anterior tip of the frog's head to the opening of the cloaca 


TL
 Total length (ML + HL + WL + PL + PPL + GL) 


WL
 Weber's length, in lateral view, measured diagonally from the inflexion on the anterior edge of the pronotum to the posterior edge of the propodeal lobe 


CI
 Cephalic index, (HW/HL) × 100 


MI
 Mandibular index, (ML/HL) × 100 


OI
 Ocular index, (EL/HW) × 100 


SI
 Scape index, (SL/HL) × 100 


## Results

### Taxonomy

#### Lenomyrmex hoelldobleri sp. n.

Taxon classificationAnimaliaHymenopteraFormicidae

http://zoobank.org/AD681140-8B64-4835-A2B7-E9730BD2CA70

Figure 1

##### Holotype worker.

ECUADOR: Esmeraldas; 4 Km SW of Alto Tambo, next to Reserve Otokiki; elevation 676 meters above sea level; GPS coordinates: 0.912306, -78.583528; 09.vii.2013; from the stomach content of a male specimen (frog voucher number: CJ1689; SVL = 36.7 mm) of the Little Devil poison frog, *Oophaga sylvatica*; leg. L. A. O'Connell, E. E. Tapia, L. A. Coloma; unique ant specimen identifier: USNMENT01124322; deposited in USNM.

##### Measurements of holotype.

HL: 1.02; HW: 0.78; ML: 0.45; SL: 0.81; EL: 0.18; WL: 1.58; PL: 0.73; PW: 0.23; PPL: 0.46; PPW: 0.35; GL: 1.00; TL: 4.77; CI: 76; OI: 23; SI 79.

##### Description, holotype worker.

Mandibles elongate, triangular with masticatory margin crenulated, 3 times longer than basal margin, sclerotized blunt peg-like denticles barely visible at 80x magnification (Fig. 1A). Clypeus without carinae, apical margin mostly convex and with a median angle; posterior margin convex, barely projects backward between frontal carinae. Frontal lobes inconspicuous, little expanded laterally, only partially covering antennal condyles. Antennal fossae large, deep, 1.5x longer than broad. Antennal scrobes absent. In full-face view, head with a broadly convex posterior cephalic margin; in full-face view, maximum width, just behind eyes, slightly narrowing posterad. Compound eyes large, protruding, with 15 facets along maximum diameter. Mesosomal profile with pronotum, mesonotum, and propodeum differentiated. Metanotal impression clearly marked (Fig. 1C). Propodeum armed with 2 long, acute spines, clearly longer than distance between their bases (Figs 1B, C). In lateral view, inferior lobes of propodeum triangular. Femora claviform. Meso- and metatibiae lacking spurs. Tarsal claws simple, elongated. In lateral view, petiole long, fusiform, pedunculate; petiolar node well-defined; antero-ventral subpetiolar process directed forward, compressed in anterior-posterior direction, giving appearance of a spine in lateral view; anterolateral edges of process continue dorsally toward sides of petiolar peduncle. In lateral view, postpetiole dome-like, lacking a ventral process.

Mandibles smooth, slightly shining (Fig. 1A). Head, mesosoma, dorsum of petiolar node and postpetiole costate. The costae longitudinal in the head frons, concentric around eyes, predominantly transverse on pronotal dorsum, transverse on mesonotum, concentrically transverse on dorsum of propodeum (Fig. 1B), longitudinal on disc of petiole and postpetiole (Figs 1B, C). Lateral margins of mesosoma with longitudinal costae, coxae with transverse costae, discrete in meso- and meta-coxae. Petiolar peduncle with granulations. Postpetiole mostly shining, and dorsolaterally with fine longitudinal striae and granulations ventrolaterally. Gaster smooth, shining except for dense punctures on pygidium and hypopygium.

Clypeal apical margin with several short, erect hairs. Head frons, leading edge of antennal scape, pronotum, node of petiole, disc of postpetiole, and gaster with scattered erect hairs, most of them longer than maximum diameter of eye (Fig. 1A & B). Erect hairs on dorsum of petiole and legs as long as, or shorter than, maximum eye diameter. Hairs on antennal scape longer than maximum diameter of antennal scape. Funicular antennal segment with numerous short decumbent hairs. Otherwise body devoid of hairs. Body black; legs and coxae lighter; antennal club, mandibles, and gastric apex yellowish-brown.

##### Distribution and ecology.

The single known specimen of *Lenomyrmex hoelldobleri* was recovered from a stomach content sample of the dendrobatid poison frog, *Oophaga sylvatica*. The habitat where the poison frog *Oophaga sylvatica* was collected was a secondary habitat with forest fragments and pastureland. The region encompasses remnant Evergreen Foothill Forests of the Western Cordillera (Ministerio del Ambiente del Ecuador 2012). This area is located in the Chocó Ecoregion, one of the most biologically diverse areas in the world with exceptionally high levels of endemism. The Chocó is considered one of the biodiversity hotspots for conservation purposes (Mittermeier et al. 1998, Myers et al. 2000) and one of the most threatened areas in the world (Brooks et al. 2002). The coastal northwest region of Ecuador, where the Alto Tambo area is found, is part of the wettest ecosystem known in Ecuador, with rainfalls ranging from 2000 up to 4000 mm annually (Ministerio del Ambiente del Ecuador 2012). Temperatures range from an annual average of 20 to 25° C (Ministerio del Ambiente del Ecuador 2012). The Foothill Forests are characterized by the dominance of tree species that can exceed 30 m in height. Trees are covered by orchids, bromeliads, ferns, and aroids. These forests have a dense herbaceous undergrowth layer dominated by Marantaceae, Araceae, and Polypodiopsida (Cerón et al. 1999). Two species of *Lenomyrmex* (*Lenomyrmex foveolatus*, *Lenomyrmex hoelldobleri*) occur in sympatry in the Alto Tambo area (Fig. 4).

##### Queen and male.

Unknown.

##### Etymology.

This species is named in honor of our colleague and friend Bert Hölldobler on the occasion of his 80^th^ birthday. Because of Bert's passion for ants, his pioneering and high-caliber contributions to entomology and behavioral ecology, as well as his dedication to mentoring the next generation of myrmecologists, myrmecology has become its own discipline in entomology, and continues to attract enthusiastic students who share Bert's love for ants.

##### Comments.

*Lenomyrmex hoelldobleri* can be distinguished from all other *Lenomyrmex* species by the following combination of character states: (i) petiolar node conspicuous, well-defined; (ii) a well-defined metanotal suture; (iii) conspicuous costae on its body; (iv) long erect hairs on the scape, and (v) size, being larger than all known species. *Lenomyrmex costatus* is morphologically most similar to *Lenomyrmex hoelldobleri* and both share the integumental sculpturing and the presence of long setae on the antennal scapes. However, *Lenomyrmex hoelldobleri* can be clearly distinguished from *Lenomyrmex costatus* by its well-defined petiolar node, the presence of the metanotal suture, its larger size, by having concentrically transverse striae on dorsum of propodeum (longitudinal in *Lenomyrmex costatus*), and the distinctly darker coloration (compare Figs 1, 2). To differentially diagnose *Lenomyrmex hoelldobleri* and *Lenomyrmex costatus*, we examined the holotype of *Lenomyrmex costatus* (Fig. 2). The specimen is deposited at Museum of Comparative Zoology at Harvard University. The specimen information is as follows: Panama; Bocas del Toro; Fortuna to Chiriqui Grande rd.; elevation 1050 meters above sea level; GPS coordinates: 8°47'N, 82°12'W; 14.vii.1987; leg. D. M. Olson (DMO523); unique ant species identifier: MCZ-ENT00036069.

#### Lenomyrmex foveolatus

Taxon classificationAnimaliaHymenopteraFormicidae

Fernández & Palacio

Figure 3

##### Gyne.

ECUADOR: Esmeraldas; Reserve Otokiki-Alto Tambo; elevation 723 meters above sea level; GPS coordinates: 0.918533, -78.566800; 08.vii.2013; from the stomach content of a female specimen (frog voucher number: CJ1658, SVL = 36.7 mm) of the Little Devil frog, *Oophaga sylvatica*; leg. L. A. O'Connell, E. E. Tapia, L. A. Coloma; unique ant specimen identifier: USNMENT01127956; deposited in USNM.

##### Gyne measurements.

HL: 0.91; HW: 0.83; ML: 0.49; SL: 0.75; EL: 0.23; WL: 1.47; PL: 0.78; PW: 0.25; PPL: 0.35; PPW: 0.29; GL: 1.41; TL: 5.40; CI: 91; MI: 55; OI: 0.29; SI 90 (n=1).

##### Description, dealate gyne.

As in the worker description (Fernández and Palacio 1999: 13–14) but mesosoma with caste-specific morphology related to wing-bearing and with the following differences: in full-face view, mid portion of anterior margin of clypeus weakly concave, forming a pair of lateral angles; compound eyes larger than in worker, with 12 ommatidia in maximum diameter; three small but conspicuous ocelli present. Dorsum of pronotum, mesoscutum, axillae, and scutellum lustrous and weakly coriaceous; dorsolateral portion of pronotum with small and sparse foveae; in dorsal view, posterior lateral portions of pronotum concave. In dorsal view, mesoscutum somewhat triangular anteriorly; parapsidal lines short, conspicuous; scuto-scutellar sulcus well-developed; posterior margin of scutellum subquadrate, lacking tubercles. Dorsum and declivity of propodeum lustrous; posterior margin of propodeum angulate, lacking tubercles or spines (as in worker). Mesopleuron clearly divided to anepisternum and katepisternum by oblique mesopleural sulcus. Pilosity of body consisting of small, simple, appressed hairs.

##### Additional material examined.

ECUADOR: Esmeraldas; Alto Tambo; elevation 788 meters above sea level; GPS coordinates: 0.907450, -78.540583; 05.vii.2013; from the stomach content of a male specimen (frog voucher number: CJ1770) of the Little Devil frog, *Oophaga sylvatica*; leg. L. A. O'Connell, E. E. Tapia, L. A. Coloma; [1w, CRC, USNMENT01127960]. Same as previous entry but, 200–300 m SW El Placer; elevation 551 meters above sea level; GPS coordinates: 0.901050, -78.618233; 07.vii.2013; from the stomach content of a male specimen (frog voucher number: CJ1632; SVL = 35.6 mm) of the Little Devil frog, *Oophaga sylvatica*; leg. L. A. O'Connell, E. E. Tapia, L. A. Coloma; [1w, QCAZ, USNMENT01127955]. Same as previous entry but, next to Reserva Otokiki (farm next to railway); elevation 676 meters above sea level; GPS coordinates: 0.912306, -78.583528; 09.vii.2013; from the stomach content of a male specimen (frog voucher number: CJ1690; SVL = 38.2 mm) of the Little Devil frog, *Oophaga sylvatica*; leg. L. A. O'Connell, E. E. Tapia, L. A. Coloma; [3w, DZUP, ICN, USNM; USNMENT01127957, USNMENT01127935, USNMENT01127958]. Same as previous entry but, from the stomach content of a female specimen (frog voucher number: CJ1691; SVL = 34.7 mm) of the Little Devil frog, *Oophaga sylvatica* [1w, QCAZ; USNMENT01127954]. Same as previous entry but, Lita; around bamboo forest; elevation 326 meters above sea level; GPS coordinates: 0.911944, -78.680833; 10.vii.2013; from the stomach content of a female specimen (frog voucher number: CJ1695; SVL = 32 mm) of the Little Devil frog, *Oophaga sylvatica*; leg. L. A. O'Connell, E. E. Tapia, L. A. Coloma; [1w, CRC, USNMENT01127936].

##### Worker measurements.

HL: 0.81–0.90; HW: 0.73–0.83; ML: 0.42–0.47; SL: 0.61–0.73; EL: 0.17–0.20; WL: 1.06–1.42; PL: 0.65–0.73; PW: 0.21–0.23; PPL: 0.29–0.35; PPW: 0.25–0.28; GL: 0.98–1.34; TL: 4.31–5.19; CI: 90–94; MI: 51–57; OI: 0.25–0.28; SI 82–95 (n=7).

##### Comments.

Specimens from the Colombian type series could not been examined, but based on the Fernández and Palacio's (1999) description, the worker specimens collected from Ecuador closely resemble the specimens from Colombia. The main differences between the specimens belonging to these two populations are: (i) the fovea on dorsum of head are scattered in the Colombian specimens and more densely clustered in the Ecuadorian individuals; (ii) the specimens from Ecuador have rounded propodeal lobes differing from the acute propodeal lobes observed in the type series from Colombia; (iii) in the specimens from Ecuador the metapleural gland bulla is striate, and striae seem absent from bulla of the Colombian specimens.

##### Distribution and ecology.

Previously only known from the type locality in western Colombia, Departamento del Valle, Darién, middle Río Calima basin. The current record near Alto Tambo extends the species geographic range 400 km south of the type locality (Fig. 4). General habitat data is the same as in the *Lenomyrmex hoelldobleri* account, except that the frog was collected in a banana plantation.

### Key to the workers of *Lenomyrmex* (modified from Delsinne and Fernández 2012)

**Table:** 

1	Mesosoma predominantly smooth and shiny, without erect hairs	**2**
–	Mesosoma with conspicuous sculpture and at least one pair of erect hairs	**3**
2(1)	Propodeum without spines; head only foveolate (SW Colombia)	***Lenomyrmex foveolatus***
–	Propodeum with a pair of acute and well-defined spines; head foveolate, with median longitudinal striae (Cordillera Oriental of the Andes in S Colombia and S Ecuador)	***Lenomyrmex inusitatus***
3(1)	Dorsum of head and petiole with longitudinal conspicuous costae; erect hairs of antennal scape as long as or longer than maximum diameter of scape	**4**
–	Dorsum of head densely rugo-reticulate; sculpture of the petiole variable, rugulate to rugo-reticulate or longitudinally striate but never costate; erect hairs of antennal scape not longer than maximum diameter of the scape	**5**
4(3)	Node of petiole inconspicuous and ill-defined; dorsum of propodeum with longitudinal striae; in dorsal view, disc of postpetiole weakly sculptured; body ferruginous yellow (W Panama)	***Lenomyrmex costatus***
–	Node of petiole conspicuous, well-defined; dorsum of propodeum with transverse striae; in dorsal view, disc of postpetiole finely striate; body black (W Ecuador)	***Lenomyrmex hoelldobleri***
5(3)	Length of propodeal spines approximately equal to distance between their bases; mesopleuron with some irregular longitudinal striae, but mostly smooth and shiny; metapleuron with irregular longitudinal striae; HL > 0.80 mm; mesosoma with only two suberect hairs on the pronotum (SW Colombia)	***Lenomyrmex mandibularis***
–	Length of propodeal spines variable, either shorter or longer than distance between their bases; metapleuron and subsequent portion of mesopleuron with fine transverse rugulae or rugo-reticulate, without smooth areas; HL < 0.80 mm; mesosoma with numerous erect to suberect hairs	**6**
6(5)	Propodeal spines shorter than distance between their bases; eyes with six or seven facets in maximum diameter; petiolar node protruding over the peduncle and well defined; postpetiolar dorsum with longitudinal striae (NW Ecuador, SW Colombia)	***Lenomyrmex wardi***
–	Propodeal spines longer than distance between their bases; eyes with about nine facets in maximum diameter; petiolar node undifferentiated from the peduncle; postpetiolar dorsum smooth and polished (Costa Rica)	***Lenomyrmex colwelli***

### Key to the known queens of *Lenomyrmex* (modified from Delsinne and Fernández 2012)

**Table:** 

1	Head foveolate; median longitudinal striae may be present. Body lacking erect hairs	**2**
–	Head densely rugo-reticulate. Body with erect hairs	**3**
2(1)	Propodeal spines present. Mesosoma shiny with sparse punctures on pronotum, mesopleuron, metapleuron, and propodeum. Scutellum and axillae foveolate, mesoscutum foveolate-striate	***Lenomyrmex inusitatus***
–	Propodeal spines absent. Mesosoma predominantly smooth and shiny, lacking punctures in mesopleuron, metapleuron, and propodeum. Pronotum with a few foveae on lateral portions. Scutellum and axillae smooth. Mesoscutum smooth and shining	***Lenomyrmex foveolatus***
3(1)	Propodeal spines approximately equal in length to distance between their bases; integument predominantly shiny; HL > 0.80	***Lenomyrmex mandibularis***
–	Propodeal spines notably shorter than distance between their bases; integument predominantly opaque; HL <0.80	***Lenomyrmex wardi***

## Discussion

All seven species of the myrmicine ant genus *Lenomyrmex* are characterized by their elongate, highly modified mandibles, which are indicative of specialized predatory habits (Fernández and Palacio 1999, Fernández 2001, Longino 2006, Delsinne and Fernández 2012). Interestingly, *Lenomyrmex* ants combine morphological characters typical of highly specialized predators with plesiotypic characters, such as the flexible suture between pronotom and mesonotum, which is atypical for myrmicine ants, but characteristic of early ant lineages with a predatory lifestyle in low-light environments (Bolton 1990, Rabeling et al. 2008, Yamane et al. 2008). This combination of plesiomorphic and derived morphological characters made it difficult to place the genus *Lenomyrmex* within the myrmicine phylogeny and its phylogenetic relationship to other members of the subfamily remained uncertain at first (Fernández and Palacio 1999). A recent molecular phylogenetic reconstruction of the subfamily Myrmicinae inferred *Lenomyrmex* as a close relative of the genus *Daceton* (Ward et al. 2015), which are predatory, arboreal ants (Wilson 1962, Azorsa and Sosa-Calvo 2008). Interestingly, the *Daceton*-species group is the sister group of the fungus-growing ants. Unfortunately, the sister-group relationship of the predatory trap-jaw ants and fungus-growing ants does not provide new insights into the much-debated evolutionary origins of the unique and highly derived fungus-growing behavior (Hölldobler and Wilson 1990, 2011, Mueller et al. 2001, Rabeling et al. 2006, Mehdiabadi and Schultz 2010). The current phylogenetic hypothesis suggests that either ant fungiculture evolved from a predatory ancestral state or, alternatively, the fungicultural and the predatory behaviors evolved along independent evolutionary trajectories from a common ancestor with generalist feeding habits. The discovery of a “missing link” would mark a real advance in our understanding about the evolutionary trajectories towards highly derived behaviors.

*Lenomyrmex* ants are rare in museum collections and the majority of the specimens have been collected sporadically in leaf-litter samples (Fernández and Palacio 1999, Fernández 2001, Longino 2006, Delsinne and Fernández 2012). So far only colonies of *Lenomyrmex mandibularis* have been collected manually because this species constructs nests in stems of a *Palicourea* species in the plant family Rubiaceae and in rotten logs (Fernández and Palacio 1999). In addition to systematic leaf litter sampling and hand collecting, the examination of stomach contents of leaf-litter foraging amphibians is a valuable source of cryptic and rarely collected ant species (Weber 1938, Delsinne and Fernández 2012, Sosa-Calvo 2015). Many species of amphibians and non-avian reptiles specialize on ant feeding and some species are predominantly myrmecophagous (Solé et al. 2002, Darst et al. 2005, Esteves et al. 2008). In the Neotropical poison frog family Dendrobatidae, myrmecophagy evolved at least twice, possibly three times independently (Santos et al. 2003, Darst et al. 2005), and the frogs sequester the skin alkaloids mostly from their ant and mite diet (McGugan et al. 2016). In addition to ants and mites, other arthropods, such as beetles and millipedes, are considered alkaloid sources for poison frogs (Dumbacher et al. 2004, Saporito et al. 2003, 2004, 2007).

To study the feeding ecology of the Little Devil poison frog, *Oophaga sylvatica*, the stomach contents of more than 300 individuals from different populations in Ecuador have been examined recently (McGugan et al. 2016, O'Connell, Sosa-Calvo et al., unpublished data). The majority of the frogs' diet consisted of ants, constituting between 40 and 86 % of diet volume in different frog populations. Of the more than 3000 examined prey items, 44 different ant genera could be identified, representing nine different subfamilies (Sosa-Calvo, O'Connell et al., unpublished data). The majority of the eaten ant genera belong to the subfamily Myrmicinae, including the rarely collected genus *Lenomyrmex*, with a total of nine specimens belonging to two species, *Lenomyrmex hoelldobleri* (the holotype worker) and *Lenomyrmex foveolatus* (seven workers and one gyne). Other cryptic and rarely collected ant genera include *Leptanilloides*, *Stigmatomma*, and *Cerapachys*, among others. To sample stomach contents of amphibians and other vertebrates solely for nutritional studies, it is not necessary to kill the animals. Stomach flushing methods have been developed and successfully applied in numerous studies, which avoids killing individuals of the study species (Solé et al. 2005). To conclude, the study of vertebrate stomach contents is not only a way of studying the trophic ecology of vertebrates themselves, but also an interesting source of cryptic and new arthropod species, including ants.

## Supplementary Material

XML Treatment for Lenomyrmex hoelldobleri

XML Treatment for Lenomyrmex foveolatus
